# Hatching the behavioral addiction egg: Reward Deficiency Solution System (RDSS)™ as a function of dopaminergic neurogenetics and brain functional connectivity linking all addictions under a common rubric

**DOI:** 10.1556/JBA.3.2014.019

**Published:** 2014-08-26

**Authors:** KENNETH BLUM, MARCELO FEBO, THOMAS MCLAUGHLIN, FRANS J. CRONJÉ, DAVID HAN, S. MARK GOLD

**Affiliations:** ^1^Department of Psychiatry and McKnight Brain Institute, University of Florida, College of Medicine, Gainesville, FL, USA; ^2^Center for Psychiatric Medicine, North Andover, MA, USA; ^3^Department of Clinical Medicine, Malibu Beach Recovery Center, Malibu Beach, CA, USA; ^4^Department of Clinical Neurology, PATH Foundation NY, NY, USA; ^5^Community Mental Health Institute, Human Global Center for Clinical & Translational Science, University of Vermont and Department of Psychiatry, University of Vermont, College of Medicine, Burlington, VT, USA; ^6^Dominion Diagnostics, Inc., North Kingstown, RI, USA; ^7^Department of Personalized Medicine, IGENE, LLC, Austin, TX, USA; ^8^University of Stellenbosch, Cape Town, South Africa; ^9^Department of Management Science and Statistics, University of Texas at San Antonio, Texas, USA

**Keywords:** neurogenetics, epigenetics, dopaminergic, Reward Deficiency Syndrome, dopamine agonist therapy

## Abstract

*Background:* Following the first association between the dopamine D2 receptor gene polymorphism and severe alcoholism, there has been an explosion of research reports in the psychiatric and behavioral addiction literature and neurogenetics. With this increased knowledge, the field has been rife with controversy. Moreover, with the advent of Whole Genome-Wide Studies (GWAS) and Whole Exome Sequencing (WES), along with Functional Genome Convergence, the multiple-candidate gene approach still has merit and is considered by many as the most prudent approach. However, it is the combination of these two approaches that will ultimately define real, genetic allelic relationships, in terms of both risk and etiology. Since 1996, our laboratory has coined the umbrella term Reward Deficiency Syndrome (RDS) to explain the common neurochemical and genetic mechanisms involved with both substance and non-substance, addictive behaviors. *Methods:* This is a selective review of peer-reviewed papers primary listed in Pubmed and Medline. *Results:* A review of the available evidence indicates the importance of dopaminergic pathways and resting-state, functional connectivity of brain reward circuits. *Discussion:* Importantly, the proposal is that the real phenotype is RDS and impairments in the brain’s reward cascade, either genetically or environmentally (epigenetically) induced, influence both substance and non-substance, addictive behaviors. Understanding shared common mechanisms will ultimately lead to better diagnosis, treatment and prevention of relapse. While, at this juncture, we cannot as yet state that we have “hatched the behavioral addiction egg”, we are beginning to ask the correct questions and through an intense global effort will hopefully find a way of “redeeming joy” and permitting *homo sapiens* live a life, free of addiction and pain.

## Introduction

Blum et al. have previously published articles on the neurogenetics of Reward Deficiency Syndrome (RDS) in terms of both substance- and non-substance-related, addictive behaviors (Blum, Oscar-Berman, Badgaiyan, Palomo & Gold, 2014). While there is extensive neurogenetic research on substance-seeking behavior, this is not the case for non-substance-related, behavioral addictions although work in this new area is growing rapidly (Demetrovics & Griffiths, 2012).

The main goal of this review is to, not only, point out the various controversies but also to demonstrate possible links between substance and non-substance, addictive behaviors. Our hope is to provide a common framework for both types of behavior, as has been the aim of the authors for almost two decades (Blum et al., 1996). This current treatise should not be considered an exhaustive review but rather a continuation of an important link in genomics and connectomics for the purpose of future, prudent addiction solutions.

Following the original work by Blum et al. (1990), which associated the *Taq-A1* allele of the dopamine D2 receptor with severe alcoholism, other researchers have reported controversial or inconsistent findings, some of which may be attributable to poor screening of controls. An example of poor screening can be seen in the work of Creemers et al. (2011), who reported negative findings relative to the role of dopaminergic gene polymorphisms in reward-seeking behavior in the Dutch general population. Although cautioned that the inclusion of subtle Reward Deficiency Syndrome (RDS) behaviors in the control group can lead to spurious results, the problem nevertheless persists to this day.

Since 1990, there have been no less than 3738 (PubMed-6-23-14) peer-reviewed articles on various peripheral and central nervous system (CNS) behaviors and physiological processes (related to addictions) on the DRD2 gene alone. Understandably, addiction or even the broader term, RDS, involves very complex gene-environment interaction. As such, one would not expect a single gene like the DRD2 to have an isolated effect. Nevertheless, and despite several negative studies, there remains a significant body of evidence positively linking the DRD2 gene polymorphism with addictive and non-addictive, reward-dependent behaviors, including those listed in Table 1.

It has been argued that the significance of the *Taq 1A* polymorphism lies in an associated decrease in neurotransmission in the nucleus accumbens leading to reward deficiency. While lower levels of striatal DAD2 receptors have been reported in imaging studies of subjects with the *Taq 1A* polymorphism, the significance of these findings is unclear. PET studies of subjects with the *Taq 1A* polymorphism have reported significantly increased striatal uptake of 18F-6FDOPA, consistent with increased DA synthesis. However, if there is increased DA synthesis and release, this may be consistent with a decrease in DAD2 receptors in response to the increased extracellular DA levels (i.e., due to a decrease in striatal D2 auto-receptors). If this theory is correct, it will contradict the surfeit theory of drug dependence. Indeed, surfeit concepts have been extended to explain escalation of cocaine abuse, claiming that the increased abuse is due to increased dopaminergic activity in the nucleus accumbens. However, recent evidence (Willuhn, Burgeno, Groblewski & Phillips, 2014) argues against this interpretation. In fact, these authors argue that the escalation of cocaine abuse is due to low dopaminergic function. Accordingly, utilizing sophisticated analyses, they argue in favor of agonistic rather than antagonistic intervention, for treating addictions.

## Problems and Controversy - Dopaminergic Surfeit or Deficit?

There is controversy about the associations between dopaminergic gene variations, such as the dopamine transporter gene (DAT) and BMI. Chen et al. (2008) had reported a significant, negative correlation between BMI and striatal DAT1 levels, however, van de Giessen et al. (2013) did not confirm this association. In this study the selection of so-called, ‘healthy’ obese subjects casts doubt on the process of screening controls for RDS behaviors. In addition, such a non-association has been reported by Thomsen et al. (2013), who also used so-called healthy obese subjects. There are, however, a number of other reports which support the DAT1 negative association with BMI (Fuemmeler et al., 2008; Need, Ahmadi, Spector & Goldstein, 2006; Sikora et al., 2013; Valomon et al., 2014; Wang et al., 2011). The negative association of DAT1 and BMI is supported by Danilovich, Mastrandrea, Cataldi and Quattrin (2014), who demonstrated that methamphetamine, known to block DAT1, reduces fat and carbohydrate intake.

Another controversy concerns the actual role of BMI as a biological marker for obesity that - as Shah and Braverman (2012) clearly pointed out - compares unfavorably with percent body fat. This conclusion was highlighted by Chen et al. (2012), whereby they found a significant correlation between carriers of the DRD2 *Taq-A1* and higher percent body fat when compared to carriers of the DRD2 *Taq-A2.*

The conclusion that sugar addiction may lead to obesity (Hone-Blanchet & Fecteau, 2014) is also controversial. However, the evidence seems to favor a bond between Substance Use Disorders, as clinically categorized in the DSM-5, and food reward (Brownell, 2012; Gold & Avena, 2013).

**Table 1.** 

Behavior	Studies that link to the DRD2 gene polymorphism
Alcohol dependence	Grzywacz, Kucharska-Mazur & Samochowiec, 2008; Munafo,Matheson & Flint, 2007; Pato, Macciardi, Pato, Verga & Kennedy, 1993; Pinto et al., 2009; Ponce et al., 2003; Smith,Watson, Gates, Ball & Foxcroft, 2008; F. Wang, Simen, Arias, Lu & Zhang, 2013; T. Y. Wang et al., 2013
Drug dependence	Al-Eitan et al., 2012; Barratt, Coller & Somogyi,2006; Chen et al., 2011; Clarke et al., 2014; Hou & Li, 2009; Jacobs et al., 2013; Lee et al., 2013; Li, Mao & Wei, 2008; Li, Ma &Beuten, 2004; Ohmoto et al., 2013; Roussotte, Jahanshad, Hibar, Thompson & for the Alzheimer’s DiseaseNeuroimaging, 2014; Schuck, Otten, Engels & Kleinjan, 2014; Sullivan et al., 2013; Suraj Singh, Ghosh &Saraswathy, 2013; Vereczkei et al., 2013; L. Wang et al., 2013; Xu et al., 2004; Young, Lawford, Nutting & Noble,2004
Mood disorders	Hettinger et al., 2012; Huertas et al., 2010; Jutras-Aswadet al., 2012; Pecina et al., 2013; Tsuchida, Nishimura & Fukui, 2012; Vaske, Makarios, Boisvert, Beaver & Wright, 2009; Whitmer& Gotlib, 2012; Zai et al., 2012; Zhang, Hu, Li, Zhang & Chen, 2014; Zhu & Shih, 1997; Zou et al., 2012
Rearing behaviors	Bakermans-Kranenburg & van Ijzendoorn, 2011; Beaver& Belsky, 2012; Masarik et al., 2014; Mills-Koonce et al., 2007
Obesity	Alsiö et al., 2014; Anitha, Abraham & Paulose, 2012; Arizaet al., 2013; Blum, Chen, Chen, Rhoades, Prihoda, Downs, Waite et al., 2008; Cameron et al., 2013; Carpenter, Wong, Li, Noble &Heber, 2013; Chen et al., 2012; Eny, Corey & El-Sohemy, 2009; Epstein, Paluch, Roemmich & Beecher,2007; Epstein et al., 2007; Fang et al., 2005; Hess et al., 2013; Huang, Yu, Zavitsanou, Han & Storlien, 2005;Jablonski, 2011; Nisoli et al., 2007; Spangler et al., 2004; Winkler et al., 2012
Motivation	Trifilieffetal., 2013
Brain metabolism	Noble, Gottschalk, Fallon, Ritchie & Wu, 1997
Pathological gambling	Gyollai et al., 2014
Attention Deficit Hyperactivity Disorder (ADHD)	Gold, Blum, Oscar-Berman & Braverman, 2014

Blum et al. (2011) discussed transfer of addiction as a potential problem associated with bariatric , and the work of Dunn et al. (2010) revealed reduced D2R availability (hypo-dopaminergic state) following bariatric surgery, suggestive of an increased requirement for self-administered drugs or behaviors linked to dopaminergic activation. Interestingly, Steele et al. (2010) found lower D2 R availability preceding bariatric surgery in five obese subjects, compared to post-surgery increased D2R levels six weeks after surgery. Increased dopamine reception would of course suggest reduced drug and/or addictive behaviors linked to enhanced dopaminergic function. However, the question is not resolved because of the findings by Dunn et al. (2010), derived from observations seven weeks after surgery, compared to six weeks by Steele et al. (2010), that found a downward trend leading again to a hypo-dopaminergic trait. The hypothesis regarding transfer of addiction seems more likely, following even longer periods post-bariatric surgery.

While there is evidence for a decreased availability of D2R in obese subjects (Volkow et al., 2009), there is some controversy that argues this is only true for severe obesity (Eisenstein et al., 2013; Kessler, Zald, Ansari, Li & Cowan, 2014). Confounding variables include control cohorts from which other RDS behaviors have not been excluded, the use of BMI as a factor may not be appropriate as a phenotype and mild obesity may not indicate the real disorder. The use of “severity” in providing a true endophenotype as discussed by a number of investigators (Blum et al., 1990; Connor, Young, Lawford, Ritchie & Noble, 2002) underscores the issue related to “mild cases” as a phenotype. Importantly, Volkow’s group has since published at least 13 papers supporting their original concept, the low D2R availability in obesity (Tomasi & Volkow, 2013). On the other hand, lowered D2R availability was not found to be associated with novelty-seeking in obesity (Savage et al., 2014).

There is evidence from Stice’s group that polymorphisms in both dopamine D2 and D4 result in a blunted response to palatable foods and subsequent weight gain (Stice & Dagher, 2010; Stice, Davis, Miller & Marti, 2008; Stice, Spoor, Bohon & Small, 2008; Stice, Spoor, Bohon, Veldhuizen & Small, 2008; Stice, Yokum, Blum & Bohon, 2010; Stice, Yokum, Bohon, Marti & Smolen, 2010; Stice, Yokum, Burger, Epstein & Smolen, 2012; Stice, Yokum, Zald & Dagher, 2011). In their later paper Stice et al. (2012) used fMRI to show that, in youth, increased striatal dopamine neurotransmission, as a co-variate, may also be a risk factor for obesity. Certainly, this supports the surfeit dopamine theory proposed by Berridge and Robinson (2000) and correctly highlights the complexity of eating disorders. An individual having increased motivation for food may fall into two categories that support either the deficit or surfeit theories, in terms of dopaminergic function. However, more research based upon both genetics and environment (epigenetics) with consideration of other variables like gender, age of onset, and in terms of “liking & wanting” may be required to understand these differences (Blum, Gardner, Oscar-Berman & Gold, 2012; Willuhn et al., 2014).

## Is there A Solution to RDS?

At this point, there is no known “cure” or magic pill for all substance and non-substance, RDS behaviors, especially, the behavioral subtypes (US FDA-approved, medical-assisted pharmaceuticals for only substance related addictions), while wrongly targeting dopamine-induced euphoria by antagonistic agents like Naltrexone and Acamprosate. Understanding the importance ofutilizing dopamine agonist therapy to treat all behavioral addictions, instead of blocking natural dopaminergic activity seems more prudent in the long-term. With supporting dopaminergic activity in mind, this laboratory has developed a complex, putative dopamine agonist, KB220Z, that has a number of very important anti-addictive effects (Blum, Chen et al., 2012). As reported in a detailed review article by Chen et al. (2011), KB220 variants have been shown to enhance brain enkephalin levels in rodents, reduce alcohol-seeking behavior in C57/BL mice and pharmacogenetically convert ethanol acceptance in preferring mice to emulate the behavior ofnon-preferring mice, such as DBA/2J.

In humans, KB220Z has been reported to reduce drug and alcohol withdrawal symptomatology exemplified by lower need for benzodiazepines, reduced days with withdrawal tremors, evidence of a lower BUD score [building up to drink] and with no severe depression detected on the Minnesota Multiphasic Personality Inventory (MMPI). Patients in group therapy had reduced stress responses, as measured by the skin conductance level, and significantly improved physical scores as well as behavioral, emotional, social and spiritual (BESS) scores. There was a six-fold decrease in Against Medical Advice (AMA) rates following detoxification, when placebo groups were compared to a KB220 variant. Healthy volunteers demonstrated enhanced focus (p300 using EEG) after taking the KB220 variant for three months. There is also evidence of reduced craving for alcohol, heroin, cocaine, and nicotine. Also, reductions in inappropriate sexual behavior and reduced post-traumatic stress (PTSD) symptoms such as paraphilia have been reported (McLaughlin et al., 2013). Quantitative electroencephalography (qEEG) studies in humans have found that KB220Z modulates theta power in anterior cingulate cortex. In abstinent heroin addicts a single dose of KB220Z compared to placebo in a pilot study (Blum, Chen, Chen, Rhoades, Prihoda, Downs, Bagchi et al., 2008) resulted in activation of the N. Accumbens (NAc) as well as activation and improvement of the prefrontal-cerebellar-occipital neural network. In addition, significantly enhanced compliance to KB220Z was found in obese patients with the DRD2 A1 allele relative to carriers of the normal compliment of DRD2 receptors using Pearson correlation (Blum, Chen, Chen, Rhoades, Prihoda, Downs, Bagchi et al., 2008) suggesting that low dopamine function equates with better outcome with KB220Z treatment.

## Genomic and Functional Mechanisms in RDS

An endeavor is underway to profoundly increase knowledge about the fundamental neural mechanisms of substance and non-substance, addictive behaviors. This task is based upon the new realization that in the mammalian brain there is complexity in the genomic networks that intimately interact with functional neural networks. Genes are under the regulatory control of epigenetic networks that may constitute a ‘code’ that shapes, and may even define, functional features of neural networks (Colvis et al., 2005). Failure at the genomic and epigenomic levels, through hereditary mechanisms or via exposure to environmental insults such as drugs of abuse, may impact the relationship between gene regulatory networks and widespread brain neural networks. Causal relationships bridging these genomic and functional levels are missing and are needed to enable effective treatments that are tailored to specific individual and population mental health diseases.

Over the past decade, novel and non-invasive functional magnetic resonance imaging (fMRI) methods have resulted in measurement of the brains intrinsic resting state activity, which is organized as functionally interrelated network states showing slow synchronous activity (Biswal, van Kylen & Hyde, 1997). Resting state functional connectivity (rsFC) is reduced in addiction to several licit and illicit drugs and in various other forms of addiction (Lu & Stein, 2014). Increased rsFC in brain reward and memory networks in both addicted human subjects and animal models was demonstrated using KB220Z, a natural dopaminergic enhancing complex. The complex developed to normalize hypodopaminergic activity referred to as RDS contains ingredients tailored to supplement the specific intermediary steps involved in neurotransmission within the brains natural reward cascade (Blum, Oscar-Berman et al., 2012). Conditions in which underlying genomic networks are altered and can negatively impact the brains intrinsic connectivity within the reward system can potentially be screened and adjusted with complex compounds such as KB220Z.

This powerful strategy can be enabled for human applications, following basic science experiments that apply high spatial-temporal resolution functional brain imaging, and genetic interrogation tools. While many laboratories across the U.S. and abroad are starting to apply optogenetic tools to examine the relationship between specific neuronal populations and disease modeling behaviors in rodents, there is a critical lack of optogenetic studies co-joined with non-invasive high field imaging.

We cannot at this time emphatically state that we have “hatched the behavioral addiction egg”. We are, however, beginning to ask the correct questions and we are encouraged by this renewed global quest for answers, so that billions of people caught up in addictive behaviors and process addictions would someday find a way of “redeeming joy” and living a life free of addiction and pain.
